# The relationship between the level of vitamin D and ruptured intracranial aneurysms

**DOI:** 10.1038/s41598-021-90760-z

**Published:** 2021-06-04

**Authors:** Sen Wei, Xin Yuan, Feng Fan, Xin‑bin Guo, Sheng Guan

**Affiliations:** 1 Department of Neurointervention, The First Affiliated Hospital of Zhengzhou University, Zhengzhou, 450052 Henan China; 2 Department of Neurology, The First Affiliated Hospital of Zhengzhou University, Zhengzhou, 450052 Henan China

**Keywords:** Neurological disorders, Cerebrovascular disorders

## Abstract

The purpose of our research is to explore whether vitamin D levels were associated with the rupture of intracranial aneurysms. In this retrospective study, 105 patients diagnosed with ruptured intracranial aneurysms (RIAs) and 185 patients diagnosed with unruptured intracranial aneurysms (UIAs) at The First Affiliated Hospital of Zhengzhou University were recruited from September 2019 to September 2020. Patients’ demographic and clinical information, including vitamin D levels, were recorded and compared. Univariate analysis showed that patients with UIAs had higher vitamin D levels than RIAs (*p* = 0.019). In addition, there were significant differences in aneurysm location (*p* < 0.001), aspirin use (*p* = 0.001), and comorbid diabetes mellitus (*p* = 0.037) between patients with UIAs and RIAs. Binary logistic regression analysis showed that the level of vitamin D was independently associated with RIAs [odds ratio (OR) 0.960; 95% confidence intervals (CI), 0.926–0.996, *p* = 0.028].

## Introduction

There is increasing evidence regarding the role of vitamin D in extra-skeletal biological functions^1,2^. Previous studies have found the relationship between vitamin D and arterial diseases^3–5^, whether it is an occlusive or aneurysmal disease, such as acute coronary syndrome, abdominal aortic aneurysm, as well as a stroke. Interestingly, low vitamin D status seems to be independent of traditional vascular risk factors by an independent effect of the vitamin D receptor (VDR) on the arterial wall^6^. Hypovitaminosis D may be involved in the processes of atherosclerosis and arterial aging, including the reduction of smooth muscle cell proliferation, resulting in vascular inflammation that can lead to damaged endothelial cells^7–9^.

Intracranial aneurysms (IAs), which are pathological dilations in major branching brain arteries, may cause serious cerebrovascular disease events, such as subarachnoid hemorrhage (SAH), a type of stroke with high disability and mortality^10^. More attention is paid to the risk factors associated with aneurysm rupture, including age, hypertension, previous SAH from another aneurysm, size and site of the aneurysm, and geographical region^11,12^. In recent years, a few studies have considered hypovitaminosis D to be connected with intracranial aneurysms^13^, but the relationship between vitamin D and intracranial aneurysm rupture is poorly understood. In this study, we explored whether vitamin D levels are associated with the rupture of intracranial aneurysms. We performed a case–control study to test the hypothesis of a positive association between vitamin D and ruptured IA (RIAs).

## Patients and methods

### Subjects

This was a retrospective case–control study, and the participants consisted of patients with intracerebral aneurysms treated between September 2019 to September 2020 at The First Affiliated Hospital of Zhengzhou University. Patients with unruptured intracranial aneurysms (UIAs) were defined as those having at least one intracranial unruptured aneurysm confirmed by imaging digital subtraction angiography (DSA), regardless of the presence of clinical symptoms. Patients with ruptured intracranial aneurysms were defined as those having aneurysmal cerebral hemorrhage, confirmed by computed tomography (CT), and were diagnosed by DSA. The exclusion criteria were as follows: (1) intake of vitamin D supplements or glucocorticoids 3 months prior; (2) patients with other intracranial vascular malformations, such as arteriovenous malformations or arteriovenous fistulas; and (3) patients with fusiform, traumatic, bacterial, or dissecting aneurysms, or SAH of unknown origin.

A total of 290 patients were included in this study. Written informed consent was obtained from all the patients before enrollment. The study was conducted in accordance with the Declaration of Helsinki and approved by the ethics committee of the First Affiliated Hospital of Zhengzhou University.

### Baseline information and clinical assessment

The following factors of the participants were recorded: age, sex, hypertension, diabetes mellitus, hyperlipidemia, heart disease, stroke, aspirin use, and the characteristics of the aneurysm (including the location and aneurysm size). In this study, the aneurysm size was equal to the maximum diameter. We also examined alcohol consumption and smoking status. Smoking and alcohol use were defined as ongoing alcohol use. We measured the levels of vitamin D, calcium and phosphorus in the morning of the first to second day after admission at the same time. Vitamin D levels were expressed by 25-hydroxy vitamin D. Blood calcium and phosphorus levels were also recorded.

### Vitamin D measurements

Quantitative determination of total 25-hydroxy (25-OH) vitamin D was performed using the electrochemical luminescence assay method on an e-602 Hitachi Roche Modular system machine (Roche Diagnostics, Cobas e602/2010/Japan, Basel, Switzerland).

### Statistical analysis

Statistical analysis was performed using SPSS software (version 23.0; SPSS, Inc. IBM Corp., Armonk, NY, USA). Numerical variables were described as the mean ± SD. Categorical variables were described by grouped data and percentages, and rates or percentages between the groups were compared with the χ^2^ test. Bivariate comparisons were performed using independent sample t-tests (continuous variables) and χ^2^ tests (categorical variables). Variables showing *p* < 0.10, in the univariate analysis, were assessed in binary logistic regression models. Age and sex (females) were also included. All confidence intervals were set at the 95% limits. Statistical significance was set at a two-tailed *p* value of < 0.05.

## Result

Demographic and clinical characteristics at baseline are shown in Table 1. This study enrolled 290 patients with IAs. There were 105 (36.21%) patients with RIAs and 185 (63.79%) with UIAs; 67.24% (n = 195) were female, and the average age was 57.19 ± 11.64 years. Age and sex were distributed homogenously between the two groups (*p* = 0.332 and *p* = 0.145, respectively). The proportion of patients with diabetes mellitus was higher in the UIA group than in the RIA group (16.22% vs. 7.62%, respectively, *p* = 0.037). Moreover, there were more patients use aspirin in the UIAs group (13.51% vs. 1.90%, respectively, *p* = 0.001). In the RIAs group, 17 (16.19%) patients had aneurysms located at the MCA, 40 (38.10%) at the ICA, 29 (27.62%) at the ACA, and 19 (18.10%) at the posterior circulation. Additionally, 37 (20.00%) aneurysms were located in the MCA, 113 (61.08%) aneurysms at the ICA, 17 (9.19%) at the ACA, and 18 (9.73%) at the posterior circulation were not ruptured. Although the proportion of ICA aneurysms was the highest in both groups, there was a significant difference (*p* < 0.001). It is worth noting that vitamin D levels were significantly higher among those with UIAs compared to those with RIAs (19.28 ± 7.91 vs. 16.86 ± 9.167, respectively, *p* = 0.019). There were no significant differences in other clinical indicators, including hypertension, smoking, alcohol use, heart disease, stroke, aneurysm size, season, and blood calcium and phosphorus status between the two groups (all *p* > 0.05).

**Table 1:** Demographic and clinical characteristics of patients with IAs at baseline.

Indicators	RIAs (n = 105)	UIAs (n = 185)	*p* value
Age, year	56.30 ± 12.38	57.69 ± 11.21	0.332
Female, n (%)	65 (61.90)	130 (70.27)	0.145
Hypertension, n (%)	59 (56.19)	93 (50.27)	0.332
Smoking, n (%)	21 (20.00)	24 (12.97)	0.112
Diabetes mellitus, n (%)	8 (7.62)	30 (16.22)	**0.037**
Hyperlipidemia, n (%)	2 (1.90)	2 (1.08)	0.957
Alcohol use, n (%)	15 (14.29)	23 (12.43)	0.653
Aspirin use, n (%)	2 (1.90)	25 (13.51)	**0.001**
Heart disease, n (%)	10 (9.52)	18 (9.73)	0.954
Stroke, n (%)	18 (17.14)	25 (13.51)	0.403
Aneurysm location, n (%)*			**< 0.001**
MCA	17 (16.19)	37 (20.00)	
ICA	40 (38.10)	113 (61.08)	
ACA	29 (27.62)	17 (9.19)	
Posterior circulation	19 (18.10)	18 (9.73)	
Aneurysm size, mm	6.12 ± 3.57	5.97 ± 4.00	0.736
Vitamin D, ng/mL	16.86 ± 9.167	19.28 ± 7.91	**0.019**
Ca, mmol/L	2.28 ± 0.10	2.28 ± 0.10	0.851
P, mmol/L	1.02 ± 0.25	2.01 ± 10.50	0.336
Season**, n (%)			0.229
Summer/autumn	42 (40.00)	61 (32.97)	
Winter/spring	63 (60.00)	124 (67.03)	

Bold: *p* < 0.05.

*MCA middle cerebral artery, *ICA* internal carotid artery, *ACA* anterior cerebral artery.

**The season of blood collection was defined as “summer/autumn” (December to May), “winter/spring” (June to November).

Table 2 summarizes the results of the binary logistic regression analysis comparing the RIAs and UIAs, which indicated that levels of vitamin D were independently associated with aneurysm rupture (OR 0.960; 95% CI 0.926–0.996, *p* = 0.028). Moreover, the use of aspirin (OR 0.083; 95% CI 0.017–0.399, *p* = 0.002) and aneurysm location (MCA, ICA, and ACA compared with aneurysms of the posterior circulation) (*p* < 0.001) were still independently associated with RIAs. Internal carotid artery location was inversely associated with RIAs (OR 0.364; 95% CI 0.168–0.788, *p* = 0.010).

**Table 2:** Binary logistic regression between UIAs and RIAs.

Indicators	*P* value	OR	95% CI
Age, year	0.401	1.010	0.987–1.034
Female, n	0.352	0.734	0.383–1.407
Vitamin D, ng/mL	**0.028**	0.960	0.926–0.996
Diabetes mellitus, n	0.248	0.590	0.241–1.444
Aspirin use, n	**0.002**	0.083	0.017–0.399
Aneurysm location, n	**< 0.001**		
MCA	0.099	0.471	0.193–1.153
ICA	**0.010**	0.364	0.168–0.788
ACA	0.125	2.142	0.810–5.667

Posterior circulation (reference).

Bold: *p* < 0.05.

## Discussion

This retrospective, case–control study suggests that vitamin D levels are independently associated with ruptured cerebral aneurysms.

Previous studies on vitamin D and RIAs are limited. In one descriptive study^13^, 25-hydroxy vitamin D levels were significantly lower in the aneurysm group than in the control group (patients without a history of cerebral aneurysm treatment). However, they did not show a significant difference in 25-hydroxy vitamin D levels between the ruptured and unruptured aneurysm subgroups. Yarelis Alvarado Reyes et al.^14^ observed 40 patients with aneurysmal subarachnoid hemorrhage (aSAH), and they found a high prevalence of vitamin D deficiency among them, but no difference in clinical outcomes (including death, vasospasm hydrocephalus requiring external ventricular drain, or infections) was observed in patients when compared with the vitamin D group. In addition, because all the experimenters were diagnosed with aSAH, they could not compare their vitamin D levels with those of patients with UIAs.

Some epidemiological studies have reported a correlation between extracranial artery dilatation, aneurysms, and vitamin D deficiency^15,16^. As Demir et al. previously reported, vitamin D deficiency is an independent factor associated with thoracic aortic dilatation (TAD) formation^17^. In an observational study of 311 elderly men with abdominal aortic aneurysm (AAA), lower vitamin D status was associated with a larger abdominal aortic aneurysm^18^. However, they did not study whether vitamin D deficiency was associated with ruptured aneurysms.

Vitamin D appears to exert anti-inflammatory effects^19^. It modulates the activation, proliferation, and differentiation of inflammatory cells through the vitamin D receptor (VDR)^20,21^. More specifically, it could reduce the production of type 1 proinflammatory cytokines (IL-12, IFN-g, IL-6, IL-8, tumor necrosis factor-a, IL-17, and IL-9) and increases the production of type 2 anti-inflammatory cytokines (IL-4, IL-5, and IL-10)^22^. Several studies have found a relationship between inflammatory response and aneurysm rupture. Researchers have found that vessel wall inflammation (arterial wall enhancement can be used as an indirect marker of vessel wall inflammation) may predict an unsteady state of intracranial aneurysms^23,24^. In addition, Hasan et al. found that the use of anti-inflammatory medications (aspirin) significantly reduced the risk of aneurysm rupture^25^. This is consistent with the results of our study. Therefore, we speculate that vitamin D may be related to aneurysm rupture by regulating the inflammatory response.

Moreover, VDR is widely distributed in the endothelium and vascular smooth muscle cells (SMCs)^26^. Decellularization or apoptosis of endothelial cells, smooth muscle cell dysfunction, and proteolysis or degradation of the extracellular matrix could result in the rupture of IAs^27–29^.

Our study had some limitations. First, this is a case–control study not allowing the establishment of any causal assumption. But, the half-life of 25-hydroxyvitamin D is about 15 days ^30^, and the patients with ruptured IAs arrived at our hospital within a few days for treatment. Therefore, we do not think the decrease of vitamin D is a consequence of the bleeding. More prospective studies are wanted to identify the relationship between them. For instance, a longitudinal, prospective cohort study with a long-term follow-up to observe clinical outcomes of patients with unruptured IAs with different levels of vitamin D. Second, several confounding variables were not considered in the present analysis, including morphological factors of IAs, eating habits, and follow-up, although these factors may play important roles in the results. Third, there were only 105 subjects in RIAs, which means it's a small sample of data. Our results also showed that the location of aneurysms was related to RIAs, but a detailed subgroup analysis was not performed.

## Conclusion

This is the first study to observe that vitamin D levels were independently associated with ruptured intracranial aneurysms. Further clinical studies with larger sample sizes should be performed to explore whether vitamin D supplementation can reduce the risk of aneurysm rupture.
